# Vector-borne pathogens in dogs from the Republic of Kosovo

**DOI:** 10.1186/s13071-025-06777-0

**Published:** 2025-04-09

**Authors:** Betim Xhekaj, Edwin Kniha, Linda Wiesmüller, Ina Hoxha, Barbara Eigner, Jovana Stefanovska, Aleksandar Cvetkovikj, Kurtesh Sherifi, Hans-Peter Fuehrer

**Affiliations:** 1 Faculty of Agriculture and Veterinary, University of Prishtina, Bulevardi “Bill Clinton”, 10000 Pristina, Kosovo; 2 Center for Pathophysiology, Infectiology and Immunology, Institute of Specific Prophylaxis and Tropical Medicine, Medical University Vienna, Kinderspitalgasse 15, 1090 Vienna, Austria; 3 Centre of Pathobiology, Institute of Parasitology, University of Veterinary Medicine Vienna, Veterinärplatz 1, 1210 Vienna, Austria; 4 Department of Parasitology and Parasitic Diseases, Faculty of Veterinary Medicine-Skopje, Ss. Cyril and Methodius University in Skopje, Lazar Pop-Trajkov 5-7, 1000 Skopje, North Macedonia

**Keywords:** Dogs, PCR, Kosovo, Vector-borne pathogens

## Abstract

**Background:**

Canine vector-borne pathogens (CVBP) are transmitted by arthropod vectors such as ticks, fleas, mosquitoes, and phlebotomine sand flies and are of global veterinary and medical importance. Dogs are important reservoir hosts, which may develop potentially life-threatening clinical signs. The Balkan area harbors diverse vector fauna and associated CVBPs, and data, particularly from the Republic of Kosovo, are scarce. Considering the high number of stray and privately owned dogs primarily kept outside, living in close contact with dogs might promote spillover of zoonotic pathogens to human populations. To combat these diseases, a One Health approach is required. Therefore, our study molecularly analyzed samples of dogs for CVBP.

**Methods:**

Blood samples of 276 dogs originating from all seven districts of Kosovo collected from 2021 to 2022 were screened using polymerase chain reaction (PCR) and sequencing for a substantial set of pathogens, including *Anaplasma* spp., *Babesia* spp., *Bartonella* spp., *Ehrlichia* spp., Filarioidea, *Hepatozoon* spp., *Mycoplasma* spp., *Rickettsia* spp., and *Trypanosoma* spp. Prevalence rates were statistically assessed on the basis of various factors such as sex, breed, age, and district.

**Results:**

In total, 150 (54.3%) dogs tested positive for at least one pathogen, comprising eight species of five genera. The most prevalent pathogens detected were *Candidatus* Mycoplasma haematoparvum (55; 19.9%), *Hepatozoon canis* (52; 18.8%), and *Mycoplasma haemocanis* (49; 17.8%). We also detected double (32; 11.6%) and triple (5; 1.8%) infections, with the latter involving combinations of *Mycoplasma* spp., *Dirofilaria repens*, *Dirofilaria immitis*, *H. canis*, or *Babesia vulpes.* In addition, prevalence rates were calculated and mapped by district. Of all included factors, significant prevalence differences were found for purebred/mixed breed dogs as well as between age groups.

**Conclusions:**

This study provides the first comprehensive polymerase chain reaction (PCR)-based screening and detection of vector-borne pathogens in dogs from Kosovo and highlights the circulation of pathogens with high veterinary importance and zoonotic potential.

**Graphical Abstract:**

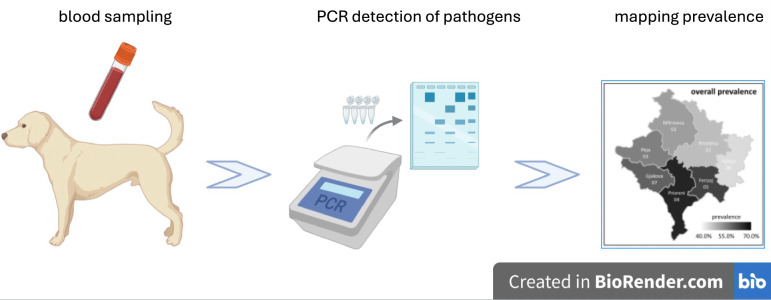

**Supplementary Information:**

The online version contains supplementary material available at 10.1186/s13071-025-06777-0.

## Background

Canine vector-borne pathogens (CVBP) comprise a wide range of globally distributed pathogens, including viruses, bacteria, protozoan parasites, and helminths that are transmitted by arthropod vectors such as ticks, fleas, mosquitoes, and phlebotomine sand flies [1]. Dogs can act as reservoir hosts and many CVBPs might develop after long incubation periods without pathognomonic clinical signs, which makes diagnosis challenging. Some also potentially cause life-threatening complications [2]. Combating these diseases requires a One Health approach as many of these pathogens have zoonotic potential, and living in close contact with dogs might promote spillover to human populations [3].

Among many others, the tick-borne pathogens *Anaplasma* spp., *Ehrlichia* spp., and *Rickettsia* spp., the mosquito-borne nematodes *Dirofilaria immitis* and *Dirofilaria repens*, as well as the sand fly-borne protozoan parasites *Leishmania* spp. have been reported to infect both humans and dogs [4–6]. In recent decades, globalization (e.g., commercial transportation, tourism, and dog travel), along with climate and environmental changes, has promoted the growth of vector populations, the shift in CVBD distribution across Europe, and their introduction to previously nonendemic areas [7].

The Balkan area harbors a diverse vector fauna; however, data on CVBD are often scant and based on heterogeneous detection methods (e.g., serology, polymerase chain reaction (PCR)-based) or completely missing for some countries [8–10]. In the Republic of Kosovo, a landlocked country in the center of the Balkans bordered by Montenegro, Serbia, Albania, and North Macedonia, data on vector-borne diseases are scarce, particularly for dogs. Particularly, the high number of stray dogs and their exports to other European countries urge for detailed understanding of CVBDs in the country. Few recent studies indicate the endemicity of important vector species and the circulation of associated diseases. The most abundant tick species, *Ixodes ricinus*, was found infected with *Anaplasma phagocytophilum*, *Babesia microti*, *Borrelia* spp., and *Rickettsia* spp. [11, 12]. In addition, antibodies against *Anaplasma*, *Borrelia*, and *Ehrlichia* in dogs have been reported by Sinani et al. in 2020 [13]. Noteworthy, data on the brown dog tick (*Rhipicephalus sanguineus* sensu lato) and its importance as a vector of CVBPs are currently lacking from Kosovo. The mosquito fauna has been investigated to comprise 13 species of six genera, and *Aedes albopictus*, the Asian tiger mosquito, was detected in 2020 in the southern part of the country for the first time [14, 15]. *Dirofilaria immitis* seropositive dogs, which had prevalence rates up to 28.6%, were observed in six of the seven districts of Kosovo [13]. Recent studies have highlighted a diverse sand fly fauna comprising nine species, with the first detection of *Leishmania infantum* in vector species and canine leishmaniasis seroprevalence in six of seven districts [16–18].

Considering the high number of stray dogs and many privately owned dogs that are primarily kept outside, further elucidation of the circulation of CVBDs, particularly those of zoonotic concern, is crucial to combat their transmission and spread. Therefore, this study aimed to analyze blood samples of dogs that have previously been subject to a canine leishmaniasis seroprevalence study [18] by targeting the detection of selected vector-borne pathogens with PCR and sequencing.

## Methods

### Study area

The present study was conducted in the Republic of Kosovo, a landlocked country in the center of the Balkan Peninsula in South-Eastern Europe. It is located between latitudes 41° and 43° N and longitudes 20° and 22° E. The study area has a continental climate with Mediterranean and Alpine influences. Kosovo is divided into seven districts according to the law of Kosovo, namely Pristina (01), Mitrovica (02), Peja (03), Prizren (04), Ferizaj (05), Gjilan (06), and Gjakova (07). In the countryside, agricultural activities include farming various animals (cattle, sheep, goats, poultry, and pigeons). Many dogs (stray, kept in private households, or shepherd dogs) are widely present.

### Dog samples and sample size

For this study, available blood samples taken in the frame of a *Leishmania* seroprevalence study in Kosovo [18] were used, which originated from dogs in private households, stray dogs (kept in shelters), and shepherd dogs. All samples were collected following the basic ethical principles and were marked by the name of the dog or chip number, location, age, breed, sex, and health status (random pathology, e.g., dermatitis, arthritis, tumor, and vasculitis).

### DNA extraction and PCR-based pathogen detection

Before DNA isolation, samples were vortexed, 200 µL blood was placed in a new tube, and 200 µL phosphate-buffered saline (PBS) was added. Thereafter, 200 µL of AL buffer was added, vortexed, and incubated at 56 °C at 550 rpm for 10 min. Then, DNA isolation was performed using the Dneasy^®^ Blood and Tissue Kit 250 (Qiagen, Hilden, Germany) following the manufacturer’s protocol with final elution in 100 µL. The DNA was stored at −20 °C until further use.

All DNA samples were tested by PCR for the presence of DNA of the following pathogens: *Anaplasma* spp., *Babesia* spp., *Bartonella* spp., *Ehrlichia* spp., Filarioidea, *Hepatozoon* spp., *Mycoplasma* spp., *Rickettsia* spp., and *Trypanosoma* spp. In addition, samples showing positive results for filarioid helminths were analyzed with species-specific PCRs for *D. immitis* and *D. repens* for inclusion of potential mixed infections. Primers and PCR protocols are presented in Table 1.

**Table 1:** PCR-based protocols for the detection of various pathogens used in this study

Organism target (length)	Primer 5′-3′	Protocol	References
Anaplasmataceae 16S rRNA (345 bp)	EHR16SD-for: GGTACCYACAGAAGAAGTCC EHR16SR-rev: TAGCACTCATCGTTTACAGC	95 °C/2 min; 35 cycles: 94 °C/1 min, 54 °C/30 s, 72 °C/30 s; 72 °C 5 min	[113]
*Babesia*^a^ 18S rRNA (700 bp)	BTH-1F: CCTGAGAAACGGCTACCACATCT BTH-1R: TTGCGACCATACTCCCCCCA	94 °C/2 min; 40 cycles: 95 °C/30 s, 68 °C/1 min, 72 °C/1 min; 72 °C 10 min	[114]
18S rRNA (561 bp)	GF2: GYYTTGTAATTGGAATGATGG GR2: CCAAAGACTTTGATTTCTCTC	94 °C/2 min; 40 cycles: 95 °C/30 s, 60 °C/1 min, 72 °C/1 min; 72 °C 10 min	[115]
*Bartonella* 16S–23S rRNA (179 bp)	bartgd_for: GATGATGATCCCAAGCCTTC B1623_rev: AACCAACTGAGCTACAAGCC	95 °C/10 min; 30 cycles: 95 °C/15 s, 60 °C/1 min, 72 °C/20 s; 72 °C 5 min	[37]
Filarioidea^b^ *cox*I (668 bp)	COlint_F: TGATTGGTGGTTTTGGTAA COlint_R: ATAAGTACGAGTATCAATATC	94 °C/2 min; 8 cycles with 0.5 °C reduction/step: 94 °C/45 s, 51 °C/45 s, 72 °C/1.5 min; 25 cycles: 94 °C/45 s, 45 °C/45 s, 72 °C/1.5 min; 72 °C 7 min	[116]
*Hepatozoon* 18S rRNA (620 bp)	H14Hepa18SFw: GAAATAACAATACAAGGCAGTTAAAATGCT H14Hepa18SRv: GTGCTGAAGGAGTCGTTTATAAAGA	95 °C/2 min; 35 cycles: 95 °C/1 min, 58 °C/1 min, 72 °C/1 min; 72 °C 7 min	[37]
*Mycoplasma* 16S rRNA (600 bp)	HBT-F: ATACGGCCCATATTCCTACG HBT-R: TGCTCCACCACTTGTTCA	94 °C/2 min; 40 cycles: 95 °C/1 min, 60 °C/1 min, 72 °C/1 min; 72 °C 7 min	[117]
*Rickettsia* 23S/5S rRNA (350–550 bp)	ITS_F: GATAGGTCGGGTGTGGAAG IST_R: TCGGGATGGGATCGTGTG	96 °C/4 min; 35 cycles: 94 °C/1 min, 52 °C/1 min, 72 °C/2 min; 72 °C 3 min	[118]
Trypanosomatidae^a^ 18S rRNA (~1320 bp)	Tryp_18S_F1: GTGGACTGCCATGGCGTTGA Tryp_18S_R1: CAGCTTGGATCTCGTCCGTTGA	96 °C/5 min; 35 cycles: 94 °C/1 min, 56 °C/1 min, 72 °C/1 min; 72 °C 5 min	[119]
18S rRNA (~960 bp)	Tryp_18S_F2: CGATGAGGCAGCGAAAAGAAATAGAG Tryp_18S_R2: GACTGTAACCTCAAAGCTTTCGCG	96 °C/5 min; 35 cycles: 94 °C/1 min, 56 °C/1 min, 72 °C/1 min; 72 °C 5 min	
*Dirofilaria immitis*^c^ COI (203 bp)	DI COI-F1: AGTGTAGAGGGTCAGCCTGAGTTA DI COI-R1: ACAGGCACTGACAATACCAAT	94 °C/2 min; 32 cycles: 94 °C/30 s, 58 °C/30 s, 72 °C/30 s; 72 °C 7 min	[120]
*Dirofilaria repens* COI (209 bp)	DR COI-F1: AGTGTTGATGGTCAACCTGAATTA DR COI-R1: GCCAAAACAGGAACAGATAAAACT	94 °C/2 min; 32 cycles: 94 °C/30 s, 58 °C/30 s, 72 °C/30 s; 72 °C 7 min	[120]

^a^Nested PCR, ^b^touchdown PCR, ^c^*Dirofilaria* discrimination for mixed infections

All PCRs were performed with either a GoTaq^®^ DNA Polymerase Master Mix (Promega, Walldorf, Germany) or a 2 × EmeraldAmp^®^ GT PCR Master Mix (Takara Bio Europe AB, Göteborg, Sweden) in a final volume of 25 µL with either an Eppendorf Mastercycler (Eppendorf AG, Hamburg, Germany) or Biometra^®^ Cycler (Analytik Jena, Jena, Germany). Bands were analyzed, cut out from the gel, and purified, as described elsewhere [16]. The samples were sent to Microsynth (Microsynth Austria GmbH, Vienna, Austria) and LGC Genomics (LGC Genomics GmbH, Berlin, Germany) for Sanger sequencing. The sequences were uploaded in the National Center for Biotechnology Information (NCBI) sequence database (accession numbers in the results section) and compared with reference sequences using the Basic Local Alignment Search Tool (BLAST) in NCBI GenBank.

### Statistical analysis and mapping of prevalences

Data were prepared with Microsoft Excel for Mac and analyzed with RStudio for Mac [19]. Categorical data were analyzed using Fisher’s exact test, using overall prevalence as a predictor variable. Odds ratios (OR) with exact 95% confidence intervals (CI) were estimated. A two-sided *P*-value < 0.05 was considered statistically significant. Prevalence was mapped with QGIS [20] using first-level administrative divisions of Kosovo (year 2015) taken from https://earthworks.stanford.edu/catalog/stanford-zh532mm5047.

## Results

### Detected pathogens

Overall, samples of 276 dogs were analyzed, comprising 138 females and 138 males from all seven districts collected in 1 year between summer 2021 and spring 2022. The mean age was 3.8 years (standard deviation (SD): 2.7 years), with the youngest dog being 4 months old and the oldest 16 years old. In total, 107 (38.8%) were purebred and 169 (61.2%) were mixed breeds. Of all dogs, 238 (86.2%) were classified as healthy and 38 (13.8%) as disrupted (random pathology unrelated to canine leishmaniasis such as dermatitis, arthritis, tumor, or vasculitis). Of the samples, 50 originated from Pristina district (01), 40 from Mitrovica (02), 35 from Peja (03), 38 from Prizren (04), 35 from Ferizaj (05), 40 from Gjilan (06), and 38 from Gjakova (07).

DNA of at least one pathogen was detected in 150 (54.3%; 95% CI 48.3–60.3) dogs, comprising eight pathogens of five genera (Table 2). Altogether, 55 (19.9%; 95% CI 15.5–25.2) DNA samples were positive for *Candidatus* Mycoplasma haematoparvum, 52 (18.8%; 95% CI 14.5–24.1) for *Hepatozoon canis*, 49 (17.8%; 95% CI 13.5–22.9) for *Mycoplasma haemocanis*, 15 (5.4%; 95% CI 3.2–9.0) for *Dirofilaria immitis*, 11 (4.0%; 95% CI 2.1–7.2) for *D. repens*, 4 (1.5%; 95% CI 0.5–3.9) for *Babesia vulpes*, 3 (1.1%; 95% CI 0.3–3.4) for *B. gibsoni*, and 1 (0.4%; 95% CI 0–2.3) for *Anaplasma phagocytophilum*. No *Bartonella* spp., *Ehrlichia* spp., *Rickettsia* spp., and *Trypanosoma* spp. DNA was detected.

**Table 2:** Detected pathogens by sex, health status, and breed

Pathogen	Sex		Health status		Breed	
	Female	Male	Normal	Disrupted	Purebred	Mixed
*A. phagocytophilum*	1 (0.7%)	–	1 (0.4%)	–	1 (0.9%)	–
*Babesia*	1 (0.7%)	6 (4.4%)	5 (2.1%)	2 (5.3%)	7 (6.5%)	–
*B. gibsoni*	1 (0.7%)	2 (1.5%)	1 (0.4%)^a^	2 (5.3%)^a^	3 (2.8%)	–
*B. vulpes*	–	4 (2.9%)	4 (1.7%)	–	4 (3.7%)	–
*Dirofilaria*	13 (9.4%)	7 (5.1%)	17 (7.1%)	3 (7.9%)	9 (8.4%)	11 (6.5%)
*D. immitis*	9 (6.5%)	6 (4.4%)	12 (5.0%)	3 (7.9%)	7 (6.5%)	8 (4.7%)
*D. repens*	9 (6.5%)	2 (1.5%)	10 (4.2%)	1 (2.6%)	6 (5.6%)	5 (3.0%)
*Hepatozoon canis*	24 (17.4%)	28 (20.3%)	45 (18.9%)	7 (18.4%)	15 (14.1%)	37 (21.9%)
*Mycoplasma*	45 (32.6%)	60 (43.5%)	90 (37.8%)	15 (39.5%)	51 (47.7%)^a^	54 (32.0%)^a^
*M. haemocanis*	24 (17.4%)	25 (18.1%)	40 (16.8%)	9 (23.7%)	18 (16.8%)	31 (18.3%)
*Candidatus* M. haematoparvum	21 (15.2%)	34 (24.6%)	50 (21.0%)	5 (13.1%)	32 (29.9%)^a^	23 (13.6%)^a^

^a^Significant difference (*P* < 0.05)

Significant differences in overall prevalence were only found for the parameters of breed and age group (Table 3). The prevalence was significantly higher in purebred compared with mixed-breed dogs (OR = 1.7, *P* = 0.04), and significantly higher prevalence rates were found in the age groups 4–6 years (OR = 2.6, *P* = 0.03), 6–8 years (OR = 4.1, *P* = 0.02), and over 8 years (OR = 2.8, *P* = 0.05) compared with the youngest dogs from the 0–2 years age group (Table 3). No significant differences were observed between sexes or health status.

**Table 3:** Overall prevalence associated with different factors

Parameter	Factor	Sample (*n*)	Positive (%)	OR (95% CI)	*P-*value
Sex	Female	138	70 (50.7%)	Reference	–
	Male	138	80 (58.0%)	1.3 (0.8–2.2)	0.28
Health Status	Disrupted	128	238 (53.8%)	Reference	–
	Normal	22	38 (57.9%)	1.2 (0.6–2.5)	0.73
Breed	Mixed	83	169 (49.1%)	Reference	–
	Purebred	67	107 (62.6%)	1.7 (1.0–2.9)^a^	0.04
Age	0–2 years	42	17 (40.5%)	Reference	–
	2–3 years	31	63 (49.2%)	1.4 (0.6–3.4)	0.43
	3–4 years	58	27 (46.6%)	1.3 (0.5–3.1)	0.68
	4–6 years	61	39 (63.9%)	2.6 (1.1–6.3)^a^	0.03
	6–8 years	23	17 (73.9%)	4.1 (1.2–15.4)^a^	0.02
	> 8 years	29	19 (65.5%)	2.8 (0.9–8.4)^a^	0.05
District	Pristina 01	50	24 (48.0%)	Reference	–
	Mitrovica 02	40	20 (50.0%)	1.1 (0.4–2.7)	1
	Peja 03	35	20 (57.1%)	1.4 (0.6–3.8)	0.51
	Prizren 04	38	26 (68.4%)	2.3 (0.9–6.3)	0.08
	Ferizaj 05	35	21 (60.0%)	1.6 (0.6–4.3)	0.38
	Gjilan 06	40	17 (42.5%)	0.8 (0.3–2.0)	0.67
	Gjakova 07	38	22 (57.9%)	1.5 (0.6–3.8)	0.4

^a^Significant difference (*P* < 0.05)

### Co-infections

**Fig. 1:**
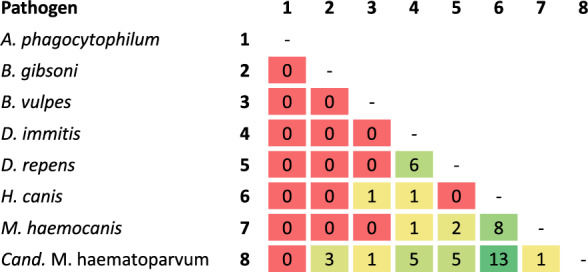
Number of detected double infections

Of all positive samples, 113 (40.9%) were single, 32 (11.6%) were double, and 5 (1.8%) were triple infections. All pathogens except *A. phagocytophilum* were associated with at least one double infection. Generally, co-infections with two pathogens were highest, including *H. canis* and *Candidatus* M. haematoparvum (13/32, 40.6%), followed by *H. canis* and *M. haemocanis* (8/32, 25.0%) and *D. immitis* and *D. repens* (6/32, 18.8%) (Fig. 1).

No significant differences in overall double infections associated with sex, health status, or breed were detected, neither between age groups nor districts.

Co-infections with three pathogens were observed in five dogs, all involving *Mycoplasma*, four *Dirofilaria*, two *H. canis*, and one *B. vulpes* (Supplementary Table 1). No significant differences between triple infections and risk factors were detected; however, a much higher but not significant triple infection rate was observed in purebred compared with mixed breeds (OR = 6.5, *P* = 0.08).

### Prevalence by district

**Fig. 2:**
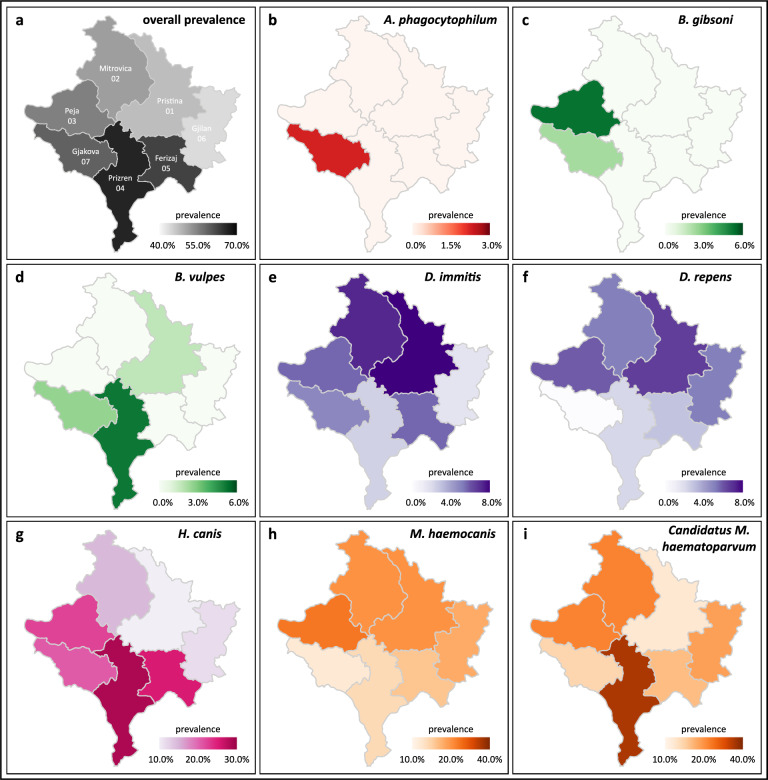
Prevalence of detected pathogens by district. Overall prevalence (**a**), *A. phagocytophilum* (**b**), *Babesia gibsoni* (**c**), *Babesia vulpes* (**d**), *Dirofilaria immitis* (**e**), *Dirofilaria repens* (**f**), *Hepatozoon canis* (**g**), *Mycoplasma haemocanis* (**h**), and *Candidatus* Mycoplasma haematoparvum (**i**)

Overall prevalence rates were highest in Prizren (68.4%) and lowest in Gjilan (42.5%) (Fig. 2). In dogs originating from Gjakova, seven of eight pathogens were detected, followed by Pristina, Peja, and Prizren with six pathogens, and Mitrovica, Ferizaj, and Gilan with five pathogens (Table 3). *Dirofilaria immits*, *H. canis*, *M. haemocanis*, and *Candidatus* M. haematoparvum were detected in all seven districts, whereas *Anaplasma phagocytophilum* was only detected in a dog from Gjakova 07.

### Pathogen typing

*Anaplasma phagocytophilum* was detected in one female dog only. The obtained sequence was identical to bacteria described in dogs from South Korea (MK239931) [21] and grey wolves (*Canis lupus*) from Germany (MN790646) [22].

DNA of *B. vulpes* (PP462098) was detected in 2.9% of the blood samples, which were identical to parasites documented in dogs from Russia (MT509981) [23]; Kyrgyzstan (OR116236) [24]; Spain [25, 26]; France [27]; and a confiscated pit bull terrier in the USA named *Babesia* sp. “Spanish dog” (EU583387) [28]. Moreover, it was examined in *Dermacentor reticulatus* in Austria [29] and raccoon dogs (*Nyctereutes procyonoides*) in Austria [30] and South Korea (OM510442). It was mainly detected in red foxes (*Vulpes vulpes*) in Austria [31, 32], Italy (KY486299), Spain (KT223483), Turkey [33], China (MW192450), the UK [34], the Czech Republic [35], Slovakia (KY175167), Croatia [36], and Bosnia and Herzegovina (KP216411) [37]. *Babesia gibsoni* (PP462099) was confirmed in three dogs, and sequences were identical to parasites described in dogs from Italy (MT752610) [38], the USA (DQ184507), Saint Kitts and Nevis (JX112784) [39], India (MN134517), Sri Lanka (OQ396762), China (CP141527), Taiwan (FJ769386), Japan (LC012808), and Myanmar (LC602469). An identical haplotype was reported from a male Boxer in Austria with travel history to Serbia, showing a co-infection with *B. canis* and *B. gibsoni* [40].

Four different haplotypes of *H. canis* (PQ836159–PQ836162) were observed. In 25 (9%) of the dogs, a haplotype (PQ855756) was found that was identical to dog samples from India (PP859411), Zambia (LC331054), Australia (MG062866) and from Eurasian golden jackals (*Canis aureus*) in Romania (KX712129; [41]) and *Ixodes holocyclus* in Australia (MG758124; [42]). Haplotype 2 (PQ836160) could be confirmed in four dogs and was identical to *H. canis* described in dogs from Cuba (MN393911), Uruguay (OR814220), and Malawi (LC169075; [43]); an Iberian wolf (*Canis lupus signatus*; PP574309) from Spain; red foxes (*V. vulpes*) in Austria (KM115969; [31]); Pampas foxes (*Lycalopex gymnocercus*) in Brazil; *C. aureus* from Austria (Mitkova et al. 2017); and *Haemaphysalis longicornis* in China and Japan (MT107096, LC169075; [44]). The third haplotype (PQ836161) was observed in two animals and was identical to pathogens in dogs in the Czech Republic (KU893127; [45]); red fox samples from Austria (KM115984, [31]) and the Czech Republic (ON128264); and raccoon (*Procyon lotor*) in the Czech Republic (OQ816791; [35]). A single sample (PQ836162) varied in one bp from the most abundant haplotype (PQ836159).

Three haplotypes of *D. repens* were documented in this study (PP552045–PP552047). Haplotype 1 (PP552045) was with one bp difference identical to findings in dogs in Italy (MT345575) [46], Slovenia (OP494254) [47], Austria (MW590257) [48], the Czech Republic (MW675691) [49], and Finland (KY828979); humans in the Czech Republic (KR998257, MW017212) [50], Slovenia (OP494269) [47], Croatia (KX265049) [51], and Finland (KY828978) [52]; and *Anopheles plumbeus* in Austria (MF695085) [53]. Haplotype 2 was identical to findings in dogs in Italy (KX265048) [51]; a human in Croatia (MT847642) [54]; and *An. daciae* in Germany (KF692102) [55]. The third haplotype was identical to findings in a dog in the Czech Republic (MW675692) [49] and humans in Italy (KT899073) [56] and Spain (MH780816) [57].

Only one haplotype (PP552044) of *D. immitis* with global distribution was documented in this study. It was identical to isolates collected in dogs from Slovenia (OP494255) [47], Hungary (KM452920) [58], Italy (AM749229, FN391553) [59], Iran (KR870344, MZ266350) [60], Bangladesh (KC107805) [61], Myanmar (ON259772) [62], Thailand (MT027229) [63], China (EU159111) [64], and Chile (OP811228) [65]. Moreover, the partial mt *COI* sequence was identical to *D. immitis* examined in grey wolf in Italy (DQ358815) [66]; golden jackal (*Canis aureus*) in Iran (MZ266360) [67]; coyote (*Canis latrans*) in the USA (ON062409) [68]; *Culex pipiens* s.l. in Spain (LC107816) [69] and Hungary (KM452924) [58]; *Cx. quinquefasciatus in* Myanmar (OL721654) [70]; domestic cat in the USA (OQ359099) [71]; and humans in Iran (MH920260) and Thailand (MW577348) [72].

*Mycoplasma haemocanis* (PQ846586) isolates were identical to samples collected in dogs in Bosnia and Herzegovina (MK107818, MK107817, MK107816), Turkey (KX641903), and also Asia and South America. Moreover, the same haplotype was documented in a red fox in Slovakia (KX752055) and cats in Brazil (KM275246, KM275242). Two haplotypes of *Candidatus* Mycoplasma haematoparvum (PQ846588, PQ846589) were documented. The haplotype (PQ846588) was identical to samples collected in dogs from Bosnia and Herzegovina (MK107815, MK107814), Italy (MH094850), Switzerland (EF416569), Romania (KY433884), and also Cuba (MZ221181), Iran (KU886262, KC762746), Iraq (PP903626), and Thailand (KT359592). Moreover, this haplotype was documented in a human with extensive animal contact (KF366443; [73]). In addition, a single sample (PQ846589) differed in one bp.

## Discussion

This study comprehensively reports, to the best of our knowledge, the first PCR-based screening and detection of vector-borne pathogens in dogs from Kosovo. Altogether, the DNA of eight pathogens of five genera was successfully amplified and sequenced. The observed overall prevalence of 54.3% highlights the circulation of various vector-borne pathogens in dogs in all districts of the country.

Of all evaluated factors, we found significantly higher infection rates in purebred dogs compared with mixed-breed dogs. Generally, genetic disorders or cancer predispositions can cluster in inbred dog populations [74, 75]. For infectious diseases, breed predispositions to disease are controversially discussed, for some infectious agents, e.g., *Leishmania infantum*, significantly higher seroprevalence rates have been found in breeds such as the Doberman Pinscher or Boxer breeds [76], while Ibizan hounds have been observed to be more resistant to *Leishmania* infections [77]. Particularly, the tick-borne pathogens *A. phagocytophilum* and *Babesia* spp. were only found in purebred dogs in our study. In contrast, Facile et al. [78] detected significantly higher infection rates and double infections of tick-borne pathogens in mixed-breed dogs than in purebreds.

In addition, all age classes above 4 years compared with 0–2 years of age showed significantly higher infection rates, being highest in the age class of 6–8 years with a prevalence of 73.9%. This is in line with other studies, particularly in endemic regions, where age correlates with the time of exposure to vectors during a dog’s life [79, 80].

We also observed higher but not significant prevalence rates in males (58%) compared with females (50.7%), which is in line with literature. Several studies on tick-borne pathogens have reported that sex is not a risk factor or a slight predisposition in male dogs, possibly related to their behavior, which exposes them to ticks more frequently [81–83].

We report the first detection of *A. phagocytophilum* by PCR in a dog from Kosovo. Only one dog originating from Gjakova was positive, resulting in a low overall prevalence of 0.4%, which is similar to a study from neighboring Albania reporting a prevalence among dogs of 1% [84]. While prevalences based on PCR and serology are generally not comparable, we would like to highlight that Sinani et al. [13] reported an *A. phagocytophilum* seroprevalence of 25% in dogs from Kosovo, which is comparable to other Balkan countries such as Croatia (4.5%) [85], Bosnia and Herzegovina (20.7%) [86], and Serbia (28.8%) [87] and confirms circulation. Discrepancies between serological and polymerase chain reaction (PCR) results are commonly reported [84, 86], as PCR is generally the most sensitive diagnostic method, but often only detects recent infections. While antibody tests can still detect past exposure through the presence of antibodies, PCR may become negative over time [88].

Two *Babesia* species were detected, namely *B. vulpes* and *B. gibsoni*, which need further discussion. To date, only one study indicated the circulation of *Babesia* in dogs from Kosovo, namely *B. canis*, the main *Babesia* species infecting dogs [89]. *Babesia vulpes*, formerly known as *Theileria annae*, primarily infects red foxes (*V. vulpes*), which most certainly represent the reservoirs, as high asymptomatic infection rates are observed regularly [90]. On the contrary, sporadic infections have been reported from dogs, and the vector involved is yet unclear; different *Ixodes* species, as well as *R. sanguineus* sensu lato, have been suspected [91–93]. *Babesia gibsoni* is a parasite of dogs, and the principal vector is *R. sanguineus* sensu lato [94]. In dogs, prevalence rates of both species are regularly low, but both small *Babesia* species are known to cause acute and chronic clinical manifestations in dogs, such as fever, lethargy, anorexia, mild-to-severe thrombocytopenia, and mild-to-severe regenerative anemia due to hemolysis, among others [95]. Noteworthy, we detected a significantly higher infection rate in dogs with disrupted health status compared with healthy dogs. In addition to our data, *B. vulpes* and *B. gibsoni* have been molecularly detected in dogs from Croatia and Serbia [95], highlighting the co-circulation of both species in Balkan countries. Thus, species identification should be applied with diagnosis in dogs and detection in ticks to evaluate potential vector species.

The detection of *Dirofilaria* DNA in dogs from all seven districts of Kosovo underlines the wide spreading of the pathogen in this region. To date, only *D. immitis* seroprevalences have been reported from dogs from Kosovo. However, *D. repens* has been reported in other Balkan countries such as Croatia, Bosnia and Herzegovina, and Serbia [96, 97]. Dirofilariosis is a disease of great veterinary importance with high zoonotic potential. *Dirofilaria repens* infections often develop asymptomatically, but nonspecific dermal alterations have been reported, such as skin nodules, pruritus, thinning, itching, and asthenia [96]. On the contrary, *D. immitis* is usually located in the heart of carnivores and causes heartworm disease (HWD) [98]. In humans, *Dirofilaria* generally does not complete its life cycle. However, few cases reported the detection of adult *D. repens*-producing microfilariae [54]. Usually, *D. repens* causes subcutaneous nodules or ocular manifestations, whereas *D. immitis* can cause pulmonary nodules [98]. Several mosquito species of different genera (*Aedes*, *Anopheles*, and *Culex*) have been implicated in the transmission of both *Dirofilaria* species [99]. In Kosovo, potential vector species, such as *Aedes vexans*, *Anopheles maculipennis* s.l., or *Culiseta annulata*, can be found. Particularly, *A. maculipennis* s.l. is highly abundant in all seven districts of Kosovo. The recent detection of *Aedes albopictus*, another potent vector, in Kosovo, might be involved in future transmission cycles if it spreads [14, 15]. Considering the frequent export of dogs from Kosovo to other (Central) European countries, the spread of dirofilariosis to previously nonendemic countries is likely and should be monitored by improved screening and preventive measures.

*Hepatozoon canis* is an apicomplexan protozoan parasite infecting domestic dogs and wild canids worldwide, and the brown dog tick, *R. sanguineus* sensu lato, serves as a vector. Transmission to the host typically happens by ingestion of ticks containing oocysts of the parasite, but vertical transmission in foxes is suspected [100, 101]. In our study, we found a *H. canis* infection rate of around 19%, similarly to other surveys reporting PCR-based prevalences in dogs of 17% in Albania [102], of 18.2% in Serbia [103], and of 26% in Hungary [104]. The infection in dogs is often subclinical but can manifest as a severe life-threatening disease with fever, cachexia, lethargy, and anemia [105], with adverse effects resulting from co-infections with other bacteria or hemoparasites [106]. We did not observe a significant difference between infection rates of healthy and disrupted dogs. However, *H. canis* presence was associated with 22 co-infections (including 2 triple infections) involving *Babesia*, *Dirofilaria*, and *Mycoplasma*, which could result in more severe clinical cases.

Similar infection rates were also detected for the hemotropic bacteria *Candidatus* Mycoplasma haematoparvum (19.9%) and *Mycoplasma haemocanis* (17.8%). Those hemotropic mycoplasmas, or hemoplasmas, are commonly known to cause chronic, subclinical infection in immunocompetent dogs but can cause hemolytic anemia in splenectomized dogs. Lower prevalence rates have been reported in Albania (8.8% for *M. haemocanis*) [84] and Greece (4.2% for *Candidatus* M. haematoparvum and 5.6% for *M. haemocanis*) [107]. The natural mode of transmission of canine haemoplasmas is currently unknown; however, *R. sanguineus* sensu lato ticks have been hypothesized as vectors owing to the usually higher prevalence rates observed in *R. sanguineus* sensu lato endemic Mediterranean countries [108, 109]. In addition, other hematophagous insects such as fleas (Siphonaptera), sucking lice (Anoplura), or keds (Hippoboscidae) have been assumed to play a role as vectors [110]. Considering the potential involvement of *R. sanguineus* sensu lato in the transmission of *Babesia*, *Hepatozoon*, and hemotropic *Mycoplasma*, the currently unclear status of this tick species in Kosovo should give rise to further studies assessing their presence in the country.

Noteworthy, the detection of DNA of vector-borne pathogens by PCR is not necessarily associated with symptoms and does not confirm active infections. In addition, we did not detect *Bartonella*, *Rickettsia*, or trypanosomatids (including *Leishmania*), probably owing to different reasons. *Bartonella* infections in dogs seem rare in Balkan countries and have been minorly addressed. For instance, Hamel et al. [102] did not detect *Bartonella* DNA in dogs from Albania, and only an infection rate of 0.7% was detected in dogs from the Czech Republic [111]. The circulation of *Rickettsia* in the Balkans seems to be evident and increasing. However, detection in dogs is underreported. In addition, the bacteria might only be detectable in blood shortly after infection, and, thus, serology or the PCR-based screening of other tissue might be more appropriate [112]. Similarly, the absence of *Leishmania* DNA in our samples can be explained. An overall canine leishmaniasis seroprevalence of 4.2% was observed among our samples in a previous study by Xhekaj et al. [18], and the endemicity of leishmaniasis is apparent in the country [16]. However, owing to the intracellular nature of the parasites, the circulation in the blood is higher shortly after infection. Generally, lymph nodes, spleens, or skin samples of infected animals should be favored, if available.

## Conclusions

For the first time, we have molecularly assessed and proven the infection of dogs with various vector-borne pathogens in the Republic of Kosovo. Our study highlights the circulation of pathogens with high veterinary importance and zoonotic potential and urges for the development of disease control strategies. High numbers of stray and shelter dogs kept outside might promote the local transmission of CVBDs. Particularly, the implementation of routine monitoring of shelter dogs (which are mostly captured stray dogs) by serology combined with PCR-based diagnosis of recent infections could be a tool to monitor and counteract the establishment of new disease transmission hotspots. However, successful countermeasures include effective treatment, if available, which can be costly and might display a limiting factor.

Given the underreported nature of vector-borne diseases in Kosovo, our results should definitely be used to raise awareness among veterinarians and serve as baseline data for further regular studies.

## Supplementary Information

Supplementary Material 1: Table 1. Triple infections by sex, health status, and breed.

## Data Availability

No datasets were generated or analyzed during the current study.

## Abbreviations

AL buffer: Lysis solution buffer
CVBD: Canine vector-borne disease
CVBP: Canine vector-borne pathogens
CI: Confidence intervals
OR: Odds ratio
PBS: Phosphate-buffered saline
rpm: Rounds per minute
PCR: Polymerase chain reaction
SD: Standard deviation
s.l.: Sensu lato

## References

[1] Otranto D, Dantas-Torres F, Breitschwerdt EB. Managing canine vector-borne diseases of zoonotic concern: part one. Trends Parasitol. 2009;25:157–63. 10.1016/j.pt.2009.01.003. 19269898 10.1016/j.pt.2009.01.003

[2] Otranto D, Dantas-Torres F, Breitschwerdt EB. Managing canine vector-borne diseases of zoonotic concern: part two. Trends Parasitol. 2009;25:228–35. 10.1016/j.pt.2009.02.005. 19346164 10.1016/j.pt.2009.02.005

[3] Day MJ. One health: the importance of companion animal vector-borne diseases. Parasit Vectors. 2011;4:49. 10.1186/1756-3305-4-49. 21489237 10.1186/1756-3305-4-49 PMC3090364

[4] Reddy MV. Human dirofilariasis: an emerging zoonosis. Trop Parasitol. 2013;3:2. 23961434 PMC3745666

[5] Rochlin I, Toledo A. Emerging tick-borne pathogens of public health importance: a mini-review. J Med Microbiol. 2020;69:781. 10.1099/JMM.0.001206. 32478654 10.1099/jmm.0.001206 PMC7451033

[6] Maia C, Conceição C, Pereira A, Rocha R, Ortuño M, Muñozid C, et al. The estimated distribution of autochthonous leishmaniasis by *Leishmania infantum* in Europe in 2005–2020. PLoS Negl Trop Dis. 2023;17:e0011497. 10.1371/JOURNAL.PNTD.0011497. 37467280 10.1371/journal.pntd.0011497 PMC10389729

[7] Beugnet F, Chalvet-Monfray K. Impact of climate change in the epidemiology of vector-borne diseases in domestic carnivores. Comp Immunol Microbiol Infect Dis. 2013;36:559–66. 10.1016/J.CIMID.2013.07.003. 23953958 10.1016/j.cimid.2013.07.003

[8] Michael L. Focus on common small animal vector-borne diseases in Central and Southeastern Europe. Acta Vet Brno. 2020;70:147–69. 10.2478/ACVE-2020-0011.

[9] Miró G, Wright I, Michael H, Burton W, Hegarty E, Rodón J, et al. Seropositivity of main vector-borne pathogens in dogs across Europe. Parasit Vectors. 2022;15:1–13. 10.1186/S13071-022-05316-5. 35668469 10.1186/s13071-022-05316-5 PMC9169295

[10] Kapo N, Zuber Bogdanović I, Gagović E, Žekić M, Veinović G, Sukara R, et al. Ixodid ticks and zoonotic tick-borne pathogens of the Western Balkans. Parasit Vectors. 2024;17:45. 10.1186/s13071-023-06116-1. 38297327 10.1186/s13071-023-06116-1 PMC10832161

[11] Sherifi K, Rexhepi A, Berxholi K, Mehmedi B, Gecaj RM, Hoxha Z, et al. Crimean-Congo hemorrhagic fever virus and *Borrelia burgdorferi* sensu lato in ticks from Kosovo and Albania. Front Vet Sci. 2018;5:38. 10.3389/fvets.2018.00038. 29560357 10.3389/fvets.2018.00038 PMC5845633

[12] Hoxha I, Xhekaj B, Halimi G, Wijnveld M, Ruivo M, Çaushi D, et al. Zoonotic tick-borne pathogens in *Ixodes ricinus* complex (Acari: Ixodidae) from urban and peri-urban areas of Kosovo. Zoonoses Pub Health. 2025;72:174–83. 10.1111/zph.13197. 39648669 10.1111/zph.13197 PMC11772905

[13] Sinani A, Aliu H, Latifi F, Haziri I, Xhekaj B, Kampen H, et al. First serological evidence of infections with selected vector-borne pathogens in dogs in Kosovo. Parasitol Res. 2020;119:3863–8. 10.1007/s00436-020-06894-y. 32974769 10.1007/s00436-020-06894-y

[14] Muja-Bajraktari N, Zhushi-Etemi F, Dikolli-Velo E, Kadriaj P, Gunay F. The composition, diversity, and distribution of mosquito fauna (Diptera: Culicidae) in Kosovo. J Vector Ecol. 2019;44:94–104. 10.1111/jvec.12333. 31124243 10.1111/jvec.12333

[15] Muja-Bajraktari N, Kadriaj P, Zhushi-Etemi F, Sherifi K, Alten B, Petric D, et al. The Asian tiger mosquito *Aedes albopictus* (Skuse) in Kosovo: first record. PLoS ONE. 2022;17:e0264300. 10.1371/JOURNAL.PONE.0264300. 35290988 10.1371/journal.pone.0264300 PMC8923454

[16] Xhekaj B, Hoxha I, Platzgummer K, Kniha E, Walochnik J, Sherifi K, et al. First Detection and molecular analysis of *Leishmania infantum* DNA in sand flies of Kosovo. Pathogens. 2023;12:1190. 10.3390/pathogens12101190. 37887706 10.3390/pathogens12101190 PMC10610191

[17] Xhekaj B, Alishani M, Rexhepi A, Jakupi X, Sherifi K. Serological survey of canine leishmaniasis in southwestern region of Kosovo. Vet Ital. 2020;56:47–50. 10.12834/VetIt.1345.7407.5. 10.12834/VetIt.1345.7407.5 32343094

[18] Xhekaj B, Stefanovska J, Sherifi K, Rexhepi A, Bizhga B, Rashikj L, et al. Seroprevalence of canine leishmaniosis in asymptomatic dogs in Kosovo. Parasitol Res. 2023;122:607–14. 10.1007/s00436-022-07762-7. 36536229 10.1007/s00436-022-07762-7

[19] Team Rs. RStudio: Integrated Development for R. RStudio, PBC, Boston, MA. 2020. http://www.rstudio.com/.

[20] QGIS Development Team. QGIS Geographic Information System. Open Source Geospatial Foundation Project. 2019. http://qgis.osgeo.org.

[21] Seo M-G, Kwon O-D, Kwak D. Molecular detection and phylogenetic analysis of canine tick-borne pathogens from Korea. Ticks Tick Borne Dis. 2020;11:101357. 10.1016/j.ttbdis.2019.101357. 31839473 10.1016/j.ttbdis.2019.101357

[22] Hodžić A, Georges I, Postl M, Duscher GG, Jeschke D, Szentiks CA, et al. Molecular survey of tick-borne pathogens reveals a high prevalence and low genetic variability of *Hepatozoon canis* in free-ranging grey wolves (*Canis lupus*) in Germany. Ticks Tick Borne Dis. 2020;11:101389. 10.1016/j.ttbdis.2020.101389. 32008999 10.1016/j.ttbdis.2020.101389

[23] Radyuk E, Karan L. A case of *Babesia vulpes* infection in a dog in Russia. Vet Parasitol Reg Stud Rep. 2020;22:100467. 10.1016/j.vprsr.2020.100467. 10.1016/j.vprsr.2020.100467 33308724

[24] Altay K, Erol U, Sahin OF, Aydin MF, Aytmirzakizi A, Dumanli N. First molecular evidence of *Babesia vogeli*, *Babesia vulpes*, and *Theileria ovis* in dogs from Kyrgyzstan. Pathogens. 2023;12:1046. 10.3390/pathogens12081046. 37624006 10.3390/pathogens12081046 PMC10460036

[25] Zahler M, Rinder H, Schein E, Gothe R. Detection of a new pathogenic *Babesia microti*-like species in dogs. Vet Parasitol. 2000;89:241–8. 10.1016/S0304-4017(00)00202-8. 10760414 10.1016/s0304-4017(00)00202-8

[26] Camacho AT, Guitian FJ, Pallas E, Gestal JJ, Olmeda AS, Goethert HK, et al. Azotemia and mortality among *Babesia microti*-like infected dogs. J Vet Intern Med. 2004;18:141–6. 10.1111/j.1939-1676.2004.tb00152.x. 15058762 10.1892/0891-6640(2004)18<141:aamabm>2.0.co;2

[27] René-Martellet M, Moro CV, Chêne J, Bourdoiseau G, Chabanne L, Mavingui P. Update on epidemiology of canine babesiosis in Southern France. BMC Vet Res. 2015;11:223. 10.1186/s12917-015-0525-3. 26303260 10.1186/s12917-015-0525-3 PMC4547427

[28] Yeagley TJ, Reichard MV, Hempstead JE, Allen KE, Parsons LM, White MA, et al. Detection of *Babesia gibsoni* and the canine small *Babesia* ‘Spanish isolate’ in blood samples obtained from dogs confiscated from dogfighting operations. J Am Vet Med Assoc. 2009;235:535–9. 10.2460/javma.235.5.535. 19719443 10.2460/javma.235.5.535

[29] Hodžić A, Zörer J, Duscher GG. Dermacentor reticulatus, a putative vector of *Babesia* cf. *microti* (syn. *Theileria annae*) piroplasm. Parasitol Res. 2017;116:1075–7. 10.1007/s00436-017-5379-0. 28116531 10.1007/s00436-017-5379-0 PMC5333375

[30] Duscher T, Hodžić A, Glawischnig W, Duscher GG. The raccoon dog (*Nyctereutes procyonoides*) and the raccoon (*Procyon lotor*)—Their role and impact of maintaining and transmitting zoonotic diseases in Austria Central Europe. Parasitol Res. 2017;116:1411–6. 10.1007/s00436-017-5405-2. 28229221 10.1007/s00436-017-5405-2 PMC5360840

[31] Duscher GG, Fuehrer H-P, Kübber-Heiss A. Fox on the run – Molecular surveillance of fox blood and tissue for the occurrence of tick-borne pathogens in Austria. Parasit Vectors. 2014;7:521. 10.1186/s13071-014-0521-7. 25413694 10.1186/s13071-014-0521-7 PMC4243377

[32] Hodžić A, Mrowietz N, Cézanne R, Bruckschwaiger P, Punz S, Habler VE, et al. Occurrence and diversity of arthropod-transmitted pathogens in red foxes (*Vulpes vulpes*) in Western Austria, and possible vertical (transplacental) transmission of Hepatozoon canis. Parasitology. 2018;145:335–44. 10.1017/S0031182017001536. 28835291 10.1017/S0031182017001536

[33] Orkun Ö, Karaer Z. Molecular characterization of *Babesia* species in wild animals and their ticks in Turkey. Infect Genet Evol. 2017;55:8–13. 10.1016/J.MEEGID.2017.08.026. 28851619 10.1016/j.meegid.2017.08.026

[34] Bartley PM, Hamilton C, Wilson C, Innes EA, Katzer F. Detection of Babesia annae DNA in lung exudate samples from Red foxes (*Vulpes vulpes*) in Great Britain. Parasit Vectors. 2016;9:84. 10.1186/s13071-016-1364-1. 26867572 10.1186/s13071-016-1364-1 PMC4751633

[35] Daněk O, Lesiczka PM, Hammerbauerova I, Volfova K, Juránková J, Frgelecová L, et al. Role of invasive carnivores (*Procyon lotor* and *Nyctereutes procyonoides*) in epidemiology of vector-borne pathogens: molecular survey from the Czech Republic. Parasit Vectors. 2023;16:219. 10.1186/s13071-023-05834-w. 37408071 10.1186/s13071-023-05834-w PMC10324142

[36] Dežđek D, Vojta L, Ćurković S, Lipej Z, Mihaljević Ž, Cvetnić Ž, et al. Molecular detection of *Theileria annae* and *Hepatozoon canis* in foxes (*Vulpes vulpes*) in Croatia. Vet Parasitol. 2010;172:333–6. 10.1016/j.vetpar.2010.05.022. 20646832 10.1016/j.vetpar.2010.05.022

[37] Hodžić A, Alić A, Fuehrer H-P, Harl J, Wille-Piazzai W, Duscher G. A molecular survey of vector-borne pathogens in red foxes (*Vulpes vulpes*) from Bosnia and Herzegovina. Parasit Vectors. 2015;8:88. 10.1186/s13071-015-0692-x. 25889961 10.1186/s13071-015-0692-x PMC4367825

[38] Carli E, De Arcangeli S, Montelli S, Caldin M, Ligorio E, Furlanello T. *Babesia gibsoni* infection in Italy: a cross sectional study of 607 blood samples belonging to dogs that needed a molecular analysis investigation (2016–2019). Vet Parasitol Reg Stud Rep. 2021;25:100596. 10.1016/j.vprsr.2021.100596. 10.1016/j.vprsr.2021.100596 34474789

[39] Loftis AD, Kelly PJ, Freeman MD, Fitzharris S, Beeler-Marfisi J, Wang C. Tick-borne pathogens and disease in dogs on St. Kitts West Indies. Vet Parasitol. 2013;196:44–9. 10.1016/J.VETPAR.2013.01.024. 23481028 10.1016/j.vetpar.2013.01.024

[40] Strobl A, Pantchev N, Martin L, Guija-De-Arespacochaga A, Hinney B, Fuehrer H-P, et al. Co-infection with *Babesia canis* and *Babesia gibsoni* in a dog. Acta Vet Hung. 2021;69:347–53. 10.1556/004.2021.00048. 10.1556/004.2021.00048 34773454

[41] Mitková B, Hrazdilová K, D’Amico G, Duscher GG, Suchentrunk F, Forejtek P, et al. Eurasian golden jackal as host of canine vector-borne protists. Parasit Vectors. 2017;10:183. 10.1186/s13071-017-2110-z. 28410591 10.1186/s13071-017-2110-z PMC5391582

[42] Greay TL, Zahedi A, Krige A-S, Owens JM, Rees RL, Ryan UM, et al. Endemic, exotic and novel apicomplexan parasites detected during a national study of ticks from companion animals in Australia. Parasit Vectors. 2018;11:197. 10.1186/s13071-018-2775-y. 29558984 10.1186/s13071-018-2775-y PMC5859549

[43] Chatanga E, Kainga H, Razemba T, Ssuna R, Swennen L, Hayashida K, et al. Molecular detection and characterization of tick-borne hemoparasites and Anaplasmataceae in dogs in major cities of Malawi. Parasitol Res. 2021;120:267–76. 10.1007/s00436-020-06967-y. 33225402 10.1007/s00436-020-06967-y

[44] Guo W-P, Xie G-C, Xue Z-Q, Yu J-J, Jian R, Du L-Y, et al. Molecular detection of *Hepatozoon canis* in dogs and ticks in Shaanxi Province China. Comp Immunol Microbiol Infect Dis. 2020;72:101514. 10.1016/j.cimid.2020.101514. 32634650 10.1016/j.cimid.2020.101514

[45] Mitková B, Hrazdilová K, Steinbauer V, D’Amico G, Mihalca AD, Modrý D. Autochthonous *Hepatozoon* infection in hunting dogs and foxes from the Czech Republic. Parasitol Res. 2016;115:4167–71. 10.1007/s00436-016-5191-2. 27431805 10.1007/s00436-016-5191-2

[46] Barlozzari G, Felice T, Salvato L, Conti R, De Liberato C, Furzi F, et al. Usual or unusual presentations of *Dirofilaria repens* in two sibling dogs: a case report. Parasitol Res. 2021;120:109–15. 10.1007/s00436-020-06926-7. 33079268 10.1007/s00436-020-06926-7 PMC7574397

[47] Kotnik T, Rataj AV, Šoba B. *Dirofilaria repens* in dogs and humans in Slovenia. J Vet Res. 2022;66:117–23. 10.2478/jvetres-2022-0008. 35582489 10.2478/jvetres-2022-0008 PMC8959679

[48] Sonnberger BW, Graf B, Straubinger RK, Rackl D, Obwaller AG, Peschke R, et al. Vector-borne pathogens in clinically healthy military working dogs in Eastern Austria. Parasitol Int. 2021;84:102410. 10.1016/j.parint.2021.102410. 34166784 10.1016/j.parint.2021.102410

[49] Juránková J, Šenkyříková Mitková B, Novotná M, Hofmannová L, Červená B, Bowman DD, et al. Further data on the distribution of *Dirofilaria* spp. in the Czech Republic in dogs. Folia Parasitol. 2022;69:2022.007. 10.14411/fp.2022.007. 10.14411/fp.2022.007 35481541

[50] Matějů J, Chanová M, Modrý D, Mitková B, Hrazdilová K, Žampachová V, et al. *Dirofilaria repens*: emergence of autochthonous human infections in the Czech Republic (case reports). BMC Infect Dis. 2016;16:171. 10.1186/s12879-016-1505-3. 27094256 10.1186/s12879-016-1505-3 PMC4837637

[51] Yilmaz E, Fritzenwanker M, Pantchev N, Lendner M, Wongkamchai S, Otranto D, et al. The mitochondrial genomes of the zoonotic canine filarial parasites *Dirofilaria* (Nochtiella) *repens* and *Candidatus Dirofilaria * (Nochtiella) *honkongensis* provide evidence for presence of cryptic species. PLoS Negl Trop Dis. 2016;10:e0005028. 10.1371/journal.pntd.0005028. 27727270 10.1371/journal.pntd.0005028 PMC5058507

[52] Pietikäinen R, Nordling S, Jokiranta S, Saari S, Heikkinen P, Gardiner C, et al. *Dirofilaria repens* transmission in Southeastern Finland. Parasit Vectors. 2017;10:561. 10.1186/s13071-017-2499-4. 29126460 10.1186/s13071-017-2499-4 PMC5681764

[53] Übleis SS, Cuk C, Nawratil M, Butter J, Schoener E, Obwaller AG, et al. Xenomonitoring of mosquitoes (Diptera: Culicidae) for the presence of filarioid helminths in Eastern Austria. Can J Infect Dis Med Microbiol. 2018;2018:1–6. 10.1155/2018/9754695. 10.1155/2018/9754695 PMC5875040 29736197

[54] Pupić-Bakrač A, Pupić-Bakrač J, Beck A, Jurković D, Polkinghorne A, Beck R. *Dirofilaria repens* microfilaremia in humans: case description and literature review. One Heal. 2021;13:100306. 10.1016/j.onehlt.2021.100306. 10.1016/j.onehlt.2021.100306 PMC8385151 34466651

[55] Kronefeld M, Kampen H, Sassnau R, Werner D. Molecular detection of *Dirofilaria immitis*, *Dirofilaria repens* and *Setaria tundr*a in mosquitoes from Germany. Parasit Vectors. 2014;7:30. 10.1186/1756-3305-7-30. 24433279 10.1186/1756-3305-7-30 PMC3898823

[56] Fontanelli Sulekova L, Gabrielli S, De Angelis M, Milardi GL, Magnani C, Di Marco B, et al. *Dirofilaria repens* microfilariae from a human node fine-needle aspirate: a case report. BMC Infect Dis. 2016;16:248. 10.1186/s12879-016-1582-3. 27266512 10.1186/s12879-016-1582-3 PMC4895828

[57] Laynez-Roldán P, Martínez-de la Puente J, Montalvo T, Mas J, Muñoz J, Figuerola J, et al. Two cases of subcutaneous dirofilariasis in Barcelona Spain. Parasitol Res. 2018;117:3679–81. 10.1007/s00436-018-6098-x. 30280219 10.1007/s00436-018-6098-x

[58] Zittra C, Kocziha Z, Pinnyei S, Harl J, Kieser K, Laciny A, et al. Screening blood-fed mosquitoes for the diagnosis of filarioid helminths and avian malaria. Parasit Vectors. 2015;8:16. 10.1186/s13071-015-0637-4. 25582219 10.1186/s13071-015-0637-4 PMC4305256

[59] Ferri E, Barbuto M, Bain O, Galimberti A, Uni S, Guerrero R, et al. Integrated taxonomy: traditional approach and DNA barcoding for the identification of filarioid worms and related parasites (Nematoda). Front Zool. 2009;6:1. 10.1186/1742-9994-6-1. 19128479 10.1186/1742-9994-6-1 PMC2657783

[60] Bamorovat M, Sharifi I, Fasihi Harandi M, Nasibi S, Sadeghi B, Khedri J, et al. Parasitological, serological and molecular study of *Dirofilaria immitis* in domestic dogs Southeastern Iran. Iran J Parasitol. 2017;12:260–6. 28761487 PMC5527037

[61] Fuehrer H-P, Treiber M, Silbermayr K, Baumann TA, Swoboda P, Joachim A, et al. Indigenous *Dirofilaria immitis* in Bangladesh. Parasitol Res. 2013;112:2393–5. 10.1007/s00436-013-3311-9. 23358737 10.1007/s00436-013-3311-9

[62] Bawm S, Khaing Y, Chel HM, Hmoon MM, Win SY, Bo M, et al. Molecular detection of *Dirofilaria immitis* and its *Wolbachia* endosymbionts in dogs from Myanmar. Curr Res Parasitol Vector-Borne Dis. 2023;4:100148. 10.1016/j.crpvbd.2023.100148. 38021190 10.1016/j.crpvbd.2023.100148 PMC10665652

[63] Colella V, Nguyen VL, Tan DY, Lu N, Fang F, Zhijuan Y, et al. Zoonotic vectorborne pathogens and ectoparasites of dogs and cats in Eastern and Southeast Asia. Emerg Infect Dis. 2020;26:1221–33. 10.3201/eid2606.191832. 32441628 10.3201/eid2606.191832 PMC7258489

[64] Huang H, Wang T, Yang G, Zhang Z, Wang C, Yang Z, et al. Molecular characterization and phylogenetic analysis of *Dirofilaria immitis* of China based on COI and 12S rDNA genes. Vet Parasitol. 2009;160:175–9. 10.1016/j.vetpar.2008.10.053. 19046806 10.1016/j.vetpar.2008.10.053

[65] Alvarez Rojas CA, Cancino-Faure B, Lillo P, Fernández ML, González AP, Ramírez AF. *Dirofilaria immitis* in dog imported from Venezuela to Chile. Emerg Infect Dis. 2023;29:439. 10.3201/eid2902.221427. 36692864 10.3201/eid2902.221427 PMC9881789

[66] Pascucci I, Fico R, Rita A, Angelo D. First notification in Italy of cardiopulmonary filariosis. Vet Ital. 2007;43:851–8. 20422563

[67] Sharifdini M, Karimi M, Ashrafi K, Soleimani M, Mirjalali H. Prevalence and molecular characterization of *Dirofilaria immitis* in road killed canids of Northern Iran. BMC Vet Res. 2022;18:161. 10.1186/s12917-022-03270-z. 35501899 10.1186/s12917-022-03270-z PMC9063217

[68] Sobotyk C, Nguyen N, Negrón V, Varner A, Saleh MN, Hilton C, et al. Detection of *Dirofilaria immitis* via integrated serological and molecular analyses in coyotes from Texas, United States. Int J Parasitol Parasites Wildl. 2022;18:20–4. 10.1016/j.ijppaw.2022.03.012. 35399590 10.1016/j.ijppaw.2022.03.012 PMC8987650

[69] Bravo-Barriga D, Parreira R, Almeida APG, Calado M, Blanco-Ciudad J, Serrano-Aguilera FJ, et al. *Culex pipiens* as a potential vector for transmission of *Dirofilaria immitis* and other unclassified Filarioidea in Southwest Spain. Vet Parasitol. 2016;223:173–80. 10.1016/j.vetpar.2016.04.030. 27198797 10.1016/j.vetpar.2016.04.030

[70] Aung ST, Bawm S, Chel HM, Htun LL, Wai SS, Eshita Y, et al. The first molecular confirmation of *Culex pipiens* complex as potential natural vectors of *Dirofilaria immitis* in Myanmar. Med Vet Entomol. 2023;37:542–9. 10.1111/mve.12652. 37017293 10.1111/mve.12652

[71] Mosley IA, Zecca IB, Tyagi N, Harvey TV, Hamer SA, Verocai GG. Occurrence of *Dirofilaria immitis* infection in shelter cats in the lower Rio Grande Valley region in South Texas, United States, using integrated diagnostic approaches. Vet Parasitol Reg Stud Rep. 2023;41:100871. 10.1016/j.vprsr.2023.100871. 10.1016/j.vprsr.2023.100871 PMC10305843 37208080

[72] Somsap Y, Boonroumkaew P, Somsap A, Rodpai R, Sadaow L, Sanpool O, et al. Ocular dirofilariasis case in Thailand confirmed by molecular analysis to be caused by *Dirofilaria immitis*. Am J Trop Med Hyg. 2022;106:204. 10.4269/AJTMH.21-0764. 10.4269/ajtmh.21-0764 PMC8733484 34634776

[73] Maggi RG, Compton SM, Trull CL, Mascarelli PE, Mozayeni BR, Breitschwerdt EB. Infection with hemotropic *Mycoplasma* species in patients with or without extensive arthropod or animal contact. J Clin Microbiol. 2013;51:3237–41. 10.1128/JCM.01125-13. 23863574 10.1128/JCM.01125-13 PMC3811635

[74] Oberbauer AM, Belanger JM, Bellumori T, Bannasch DL, Famula TR. Ten inherited disorders in purebred dogs by functional breed groupings. Canine Genet Epidemiol. 2015;2:9. 10.1186/s40575-015-0021-x. 26401337 10.1186/s40575-015-0021-x PMC4579393

[75] Dobson JM. Breed-predispositions to cancer in pedigree dogs. ISRN Vet Sci. 2013;2013:1–23. 10.1155/2013/941275. 10.1155/2013/941275 PMC3658424 23738139

[76] Edo M, Marín-García PJ, Llobat L. Is the prevalence of *Leishmania infantum* linked to breeds in dogs? Characterization of seropositive dogs in Ibiza. Animals. 2021;11:2579. 10.3390/ani11092579. 34573545 10.3390/ani11092579 PMC8466328

[77] Solano-Gallego L, Llull J, Ramos G, Riera C, Arboix M, Alberola J, et al. The Ibizian hound presents a predominantly cellular immune response against natural *Leishmania* infection. Vet Parasitol. 2000;90:37–45. 10.1016/S0304-4017(00)00223-5. 10828510 10.1016/s0304-4017(00)00223-5

[78] Facile V, Sabetti MC, Balboni A, Urbani L, Tirolo A, Magliocca M, et al. Detection of *Anaplasma* spp. and *Ehrlichia* spp. in dogs from a veterinary teaching hospital in Italy: a retrospective study 2012–2020. Vet Res Commun. 2024;48:1727–40. 10.1007/s11259-024-10358-4. 38536514 10.1007/s11259-024-10358-4 PMC11147850

[79] Ebani VV, Bertelloni F, Turchi B, Cerri D. Serological and molecular survey of *Anaplasma phagocytophilum* in Italian hunting dogs. Ann Agric Environ Med. 2013;20:289–92. 23772578

[80] Dzięgiel B, Adaszek Ł, Carbonero A, Łyp P, Winiarczyk M, Dębiak P, et al. Detection of canine vector-borne diseases in Eastern Poland by ELISA and PCR. Parasitol Res. 2016;115:1039–44. 10.1007/s00436-015-4832-1. 26581374 10.1007/s00436-015-4832-1 PMC4759218

[81] Sainz Á, Roura X, Miró G, Estrada-Peña A, Kohn B, Harrus S, et al. Guideline for veterinary practitioners on canine ehrlichiosis and anaplasmosis in Europe. Parasit Vectors. 2015;8:75. 10.1186/s13071-015-0649-0. 25649069 10.1186/s13071-015-0649-0 PMC4324656

[82] Ebani VV. Serological survey of *Ehrlichia canis* and *Anaplasma phagocytophilum* in dogs from Central Italy: an update (2013–2017). Pathogens. 2019;8:3. 10.3390/pathogens8010003. 30621134 10.3390/pathogens8010003 PMC6471581

[83] Hazelrig CM, Gettings JR, Cleveland CA, Varela-Stokes A, Majewska AA, Hubbard K, et al. Spatial and risk factor analyses of vector-borne pathogens among shelter dogs in the Eastern United States. Parasit Vectors. 2023;16:197. 10.1186/s13071-023-05813-1. 37301970 10.1186/s13071-023-05813-1 PMC10257847

[84] Hamel D, Shukullari E, Rapti D, Silaghi C, Pfister K, Rehbein S. Parasites and vector-borne pathogens in client-owned dogs in Albania. Blood pathogens and seroprevalences of parasitic and other infectious agents. Parasitol Res. 2016;115:489–99. 10.1007/s00436-015-4765-8. 26453093 10.1007/s00436-015-4765-8

[85] Jurković D, Beck A, Huber D, Mihaljević Ž, Polkinghorne A, Martinković F, et al. Seroprevalence of vector-borne pathogens in dogs from Croatia. Parasitol Res. 2019;118:347–52. 10.1007/s00436-018-6129-7. 30377795 10.1007/s00436-018-6129-7

[86] Maksimović Z, Dervišević M, Zahirović A, Rifatbegović M. Seroprevalence of *Anaplasma* spp. and *Ehrlichia* spp. and molecular detection of *Anaplasma phagocytophilum* and *Anaplasma platys* in stray dogs in Bosnia and Herzegovina. Ticks Tick Borne Dis. 2022;13:101875. 10.1016/j.ttbdis.2021.101875. 34894522 10.1016/j.ttbdis.2021.101875

[87] Kovačević Filipović MM, Beletić AD, Ilić Božović AV, Milanović Z, Tyrrell P, Buch J, et al. Molecular and serological prevalence of *Anaplasma phagocytophilum*, *A*. *platys*, *Ehrlichia canis*, *E*. *chaffeenses*, *E*. *ewingii*, *Borrelia burgdorferi*, *Babesia canis*, *B*. *gibsoni* and *B*. *vogeli* among clinically healthy outdoor dogs in Serbia. Vet Parasitol Reg Stud Rep. 2018;14:117–22. 10.1016/j.vprsr.2018.10.001. 10.1016/j.vprsr.2018.10.001 31014716

[88] Schäfer I, Kohn B, Silaghi C, Fischer S, Marsboom C, Hendrickx G, et al. Molecular and serological detection of *Anaplasma phagocytophilum* in dogs from Germany (2008–2020). Animals. 2023;13:720. 10.3390/ani13040720. 36830507 10.3390/ani13040720 PMC9952382

[89] Lazri T, Duscher G, Edelhofer R, Bytyci B, Gjino P, Joachim A. Arthropod-borne parasites of dogs, especially *Leishmania*, in the Kosovo and Albania. Wien Klin Wochenschr. 2008;120:54–8. 10.1007/s00508-008-1076-4. 19066774 10.1007/s00508-008-1076-4

[90] Baneth G, Florin-Christensen M, Cardoso L, Schnittger L. Reclassification of *Theileria annae* as *Babesia vulpes* sp. nov. Parasit Vectors. 2015;8:207. 10.1186/s13071-015-0830-5. 25890372 10.1186/s13071-015-0830-5 PMC4393874

[91] Iori A, Gabrielli S, Calderini P, Moretti A, Pietrobelli M, Tampieri MP, et al. Tick reservoirs for piroplasms in Central and Northern Italy. Vet Parasitol. 2010;170:291–6. 10.1016/j.vetpar.2010.02.027. 20304560 10.1016/j.vetpar.2010.02.027

[92] Lledó L, Giménez-Pardo C, Domínguez-Peñafiel G, Sousa R, Gegúndez MI, Casado N, et al. Molecular detection of hemoprotozoa and *Rickettsia* species in arthropods collected from wild animals in the Burgos Province Spain. Vector-Borne Zoonotic Dis. 2010;10:735–8. 10.1089/vbz.2009.0114. 20055580 10.1089/vbz.2009.0114

[93] Najm N-A, Meyer-Kayser E, Hoffmann L, Herb I, Fensterer V, Pfister K, et al. A molecular survey of *Babesia* spp. and *Theileria* spp. in red foxes (*Vulpes vulpes*) and their ticks from Thuringia Germany. Ticks Tick Borne Dis. 2014;5:386–91. 10.1016/j.ttbdis.2014.01.005. 24717451 10.1016/j.ttbdis.2014.01.005

[94] Teodorowski O, Kalinowski M, Winiarczyk D, Dokuzeylül B, Winiarczyk S, Adaszek Ł. *Babesia gibsoni* Infection in dogs—A European perspective. Animals. 2022;12:730. 10.3390/ani12060730. 35327127 10.3390/ani12060730 PMC8944637

[95] Solano-Gallego L, Sainz Á, Roura X, Estrada-Peña A, Miró G. A review of canine babesiosis: the European perspective. Parasit Vectors. 2016;9:336. 10.1186/s13071-016-1596-0. 27289223 10.1186/s13071-016-1596-0 PMC4902949

[96] Capelli G, Genchi C, Baneth G, Bourdeau P, Brianti E, Cardoso L, et al. Recent advances on *Dirofilaria repens* in dogs and humans in Europe. Parasit Vectors. 2018;11:663. 10.1186/S13071-018-3205-X. 30567586 10.1186/s13071-018-3205-x PMC6299983

[97] Tasić-Otašević S, Savić S, Jurhar-Pavlova M, Stefanovska J, Stalević M, Ignjatović A, et al. Molecular survey of *Dirofilaria* and *Leishmania* species in dogs from Central Balkan. Animals. 2022;12:911. 10.3390/ani12070911. 35405899 10.3390/ani12070911 PMC8997140

[98] Méndez JC, Carretón E, Martínez S, Tvarijonaviciute A, Cerón JJ, Montoya-Alonso JA. Acute phase response in dogs with *Dirofilaria immitis*. Vet Parasitol. 2014;204:420–5. 10.1016/J.VETPAR.2014.05.016. 24893697 10.1016/j.vetpar.2014.05.016

[99] Simón F, Siles-Lucas M, Morchón R, González-Miguel J, Mellado I, Carretón E, et al. Human and animal dirofilariasis: the emergence of a zoonotic mosaic. Clin Microbiol Rev. 2012;25:507–44. 10.1128/CMR.00012-12. 22763636 10.1128/CMR.00012-12 PMC3416488

[100] Baneth G, Samish M, Shkap V. Life cycle of *Hepatozoon canis* (Apicomplexa: Adeleorina: Hepatozoidae) in the tick *Rhipicephalus sanguineus* and domestic dog (*Canis familiaris*). J Parasitol. 2007;93:283–99. 10.1645/GE-494R.1. 17539411 10.1645/GE-494R.1

[101] Baneth G. Perspectives on canine and feline hepatozoonosis. Vet Parasitol. 2011;181:3–11. 10.1016/j.vetpar.2011.04.015. 21620568 10.1016/j.vetpar.2011.04.015

[102] Hamel D, Silaghi C, Knaus M, Visser M, Kusi I, Rapti D, et al. Detection of *Babesia**canis* subspecies and other arthropod-borne diseases in dogs from Tirana, Albania. Wien Klin Wochenschr. 2009;121:42–5. 10.1007/s00508-009-1234-3. 19915816 10.1007/s00508-009-1234-3

[103] Sukara R, Andrić N, Andrić JF, Mihaljica D, Veinović G, Ranković V, et al. Autochthonous infection with *Ehrlichia canis* and *Hepatozoon canis* in dogs from Serbia. Vet Med Sci. 2023;9:111–8. 10.1002/vms3.1061. 36580396 10.1002/vms3.1061 PMC9857103

[104] Hornok S, Tánczos B, Fernández de Mera IG, de la Fuente J, Hofmann-Lehmann R, Farkas R. High prevalence of *Hepatozoon*-infection among shepherd dogs in a region considered to be free of *Rhipicephalus sanguineus*. Vet Parasitol. 2013;196:189–93. 10.1016/j.vetpar.2013.02.009. 23499483 10.1016/j.vetpar.2013.02.009

[105] Baneth G, Weigler B. Retrospective case-control study of hepatozoonosis in dogs in Israel. J Vet Intern Med. 1997;11:365–70. 10.1111/j.1939-1676.1997.tb00482.x. 9470163 10.1111/j.1939-1676.1997.tb00482.x

[106] Uiterwijk M, Vojta L, Šprem N, Beck A, Jurković D, Kik M, et al. Diversity of *Hepatozoon* species in wild mammals and ticks in Europe. Parasit Vectors. 2023;16:27. 10.1186/s13071-022-05626-8. 36694253 10.1186/s13071-022-05626-8 PMC9872412

[107] Tennant KV, Barker EN, Polizopoulou Z, Helps CR, Tasker S. Real-time quantitative polymerase chain reaction detection of haemoplasmas in healthy and unhealthy dogs from Central Macedonia Greece. J Small Anim Pract. 2011;52:645–9. 10.1111/j.1748-5827.2011.01126.x. 22017540 10.1111/j.1748-5827.2011.01126.x

[108] Barker EN, Tasker S, Day MJ, Warman SM, Woolley K, Birtles R, et al. Development and use of real-time PCR to detect and quantify *Mycoplasma haemocanis* and “*Candidatus Mycoplasma haematoparvum*” in dogs. Vet Microbiol. 2010;140:167–70. 10.1016/j.vetmic.2009.07.006. 19646827 10.1016/j.vetmic.2009.07.006 PMC2805721

[109] Novacco M, Meli ML, Gentilini F, Marsilio F, Ceci C, Pennisi MG, et al. Prevalence and geographical distribution of canine hemotropic *Mycoplasma* infections in Mediterranean countries and analysis of risk factors for infection. Vet Microbiol. 2010;142:276–84. 10.1016/j.vetmic.2009.09.069. 19931320 10.1016/j.vetmic.2009.09.069

[110] Beus K, Goudarztalejerdi A, Sazmand A. Molecular detection and identification of hemotropic *Mycoplasma* species in dogs and their ectoparasites in Iran. Sci Rep. 2024;14:580. 10.1038/s41598-024-51173-w. 38182649 10.1038/s41598-024-51173-w PMC10770070

[111] Konvalinová J, Svobodová V, Molinková D, Svoboda M. PCR detection of *Bartonella* spp. in the dog. Acta Vet Brno. 2014;83:79–82. 10.2754/avb201483020079.

[112] Abdad MY, Abou Abdallah R, Fournier P-E, Stenos J, Vasoo S. A Concise Review of the epidemiology and diagnostics of rickettsioses: *Rickettsia* and *Orientia* spp. J Clin Microbiol. 2018. 10.1128/JCM.01728-17. 29769278 10.1128/JCM.01728-17 PMC6062794

[113] Parola P, Roux V, Camicas JL, Baradji I, Brouqui P, Raoult D. Detection of Ehrlichiae in African ticks by polymerase chain reaction. Trans R Soc Trop Med Hyg. 2000;94:707–8. 10.1016/S0035-9203(00)90243-8. 11198664 10.1016/s0035-9203(00)90243-8

[114] Zintl A, Finnerty EJ, Murphy TM, de Waal T, Gray JS. Babesias of red deer (*Cervus elaphus*) in Ireland. Vet Res. 2011;42:7. 10.1186/1297-9716-42-7. 21314977 10.1186/1297-9716-42-7 PMC3037898

[115] Bonnet S, Jouglin M, Malandrin L, Becker C, Agoulon A, L’Hostis M, et al. Transstadial and transovarial persistence of *Babesia divergens* DNA in *Ixodes ricinus* ticks fed on infected blood in a new skin-feeding technique. Parasitology. 2007;134:197–207. 10.1017/S0031182006001545. 17076925 10.1017/S0031182006001545

[116] Hamer GL, Anderson TK, Berry GE, Makohon-Moore AP, Crafton JC, Brawn JD, et al. Prevalence of filarioid nematodes and trypanosomes in American robins and house sparrows, Chicago USA. Int J Parasitol Parasites Wildl. 2013;2:42–9. 10.1016/J.IJPPAW.2012.11.005. 24533314 10.1016/j.ijppaw.2012.11.005 PMC3862512

[117] Criado-Fornelio A, Martinez-Marcos A, Buling-Saraña A, Barba-Carretero JC. Presence of *Mycoplasma haemofelis*, *Mycoplasma haemominutum* and piroplasmids in cats from southern Europe: a molecular study. Vet Microbiol. 2003;93:307–17. 10.1016/S0378-1135(03)00044-0. 12713893 10.1016/s0378-1135(03)00044-0

[118] Vitorino L, Zé-Zé L, Sousa A, Bacellar F, Tenreiro R. rRNA Intergenic spacer regions for phylogenetic analysis of *Rickettsia* species. Ann N Y Acad Sci. 2003;990:726–33. 10.1111/J.1749-6632.2003.TB07451.X. 12860714 10.1111/j.1749-6632.2003.tb07451.x

[119] Peña-Espinoza M, Em D, Shahi-Barogh B, Berer D, Duscher GG, van der Vloedt L, et al. Molecular pathogen screening of louse flies (Diptera: Hippoboscidae) from domestic and wild ruminants in Austria. Parasit Vectors. 2023;16:179. 10.1186/s13071-023-05810-4. 37269018 10.1186/s13071-023-05810-4 PMC10236838

[120] Rishniw M, Barr SC, Simpson KW, Frongillo MF, Franz M, Dominguez Alpizar JL. Discrimination between six species of canine microfilariae by a single polymerase chain reaction. Vet Parasitol. 2006;135:303–14. 10.1016/j.vetpar.2005.10.013. 16289566 10.1016/j.vetpar.2005.10.013

