# Supplementary material for: Vector-borne pathogens in dogs from the Republic of Kosovo
Source: Parasit Vectors. 2025 Apr 9;18:136. doi: 10.1186/s13071-025-06777-0 (PMC11983783; doi:10.1186/s13071-025-06777-0)
Supplement: Supplementary file 1 — Supplementary Material 1: Table 1. Triple infections by sex, health status, and breed. [file 13071_2025_6777_MOESM1_ESM.docx]

**Supplementary Table 1.** Triple infections by sex, health status, and breed.

|  | **sex** | |  | **health status** | |  | **breed** | | |
| --- | --- | --- | --- | --- | --- | --- | --- | --- | --- |
| **co-infection** | female | male |  | normal | disrupted |  | purebred | mixed |  |
| ***B. vulpes* +**  ***H. canis* +**  ***Cand.* M. haematoparvum** | - | 1 (0.7%) |  | 1 (0.4%) | - |  | 1 (0.9%) | - |  |
| ***D. immitis* +**  ***D. repens* +**  ***Cand.* M. haematoparvum** | 2 (1.5%) | 1 (0.7%) |  | 3 (1.3%) | - |  | 3 (2.8%) | - |  |
| ***D. immits* +**  ***H. canis* +**  ***M. haemocanis*** | - | 1 (0.7%) |  | 1 (0.4%) | - |  | - | 1 (0.6%) |  |
